# Recovery of Apraxia of Speech and Aphasia in Patients With Hand Motor Impairment After Stroke

**DOI:** 10.3389/fneur.2021.634065

**Published:** 2021-03-31

**Authors:** Helena Hybbinette, Ellika Schalling, Jeanette Plantin, Catharina Nygren-Deboussard, Marika Schütz, Per Östberg, Påvel G. Lindberg

**Affiliations:** ^1^Department of Clinical Science, Intervention and Technology, Division of Speech and Language Pathology, Karolinska Institutet, Stockholm, Sweden; ^2^Department of Clinical Sciences, Danderyd Hospital, Division of Rehabilitation Medicine, Stockholm, Sweden; ^3^Department of Rehabilitation Medicine, Danderyd University Hospital, Stockholm, Sweden; ^4^Medical Unit Speech and Language Pathology, Karolinska University Hospital, Stockholm, Sweden; ^5^Institut de Psychiatrie et Neurosciences Paris, Inserm U1266, Université de Paris, Paris, France

**Keywords:** stroke, recovery, apraxia of speech, aphasia (language), hand motor impairment, prevalence

## Abstract

**Objective:** Aphasia and apraxia of speech (AOS) after stroke frequently co-occur with a hand motor impairment but few studies have investigated stroke recovery across motor and speech-language domains. In this study, we set out to test the shared recovery hypothesis. We aimed to (1) describe the prevalence of AOS and aphasia in subacute stroke patients with a hand motor impairment and (2) to compare recovery across speech-language and hand motor domains. In addition, we also explored factors predicting recovery from AOS.

**Methods:** Seventy participants with mild to severe paresis in the upper extremity were assessed; 50% of these (*n* = 35) had left hemisphere (LH) lesions. Aphasia, AOS and hand motor assessments and magnetic resonance imaging were conducted at 4 weeks (A1) and at 6 months (A2) after stroke onset. Recovery was characterized in 15 participants showing initial aphasia that also had complete follow-up data at 6 months.

**Results:** All participants with AOS and/or aphasia had LH lesions. In LH lesioned, the prevalence of aphasia was 71% and of AOS 57%. All participants with AOS had aphasia; 80% of the participants with aphasia also had AOS. Recovery in aphasia (*n* = 15) and AOS (*n* = 12) followed a parallel pattern to that observed in hand motor impairment and recovery correlated positively across speech-language and motor domains. The majority of participants with severe initial aphasia and AOS showed a limited but similar amount of recovery across domains. Lesion volume did not correlate with results from behavioral assessments, nor with recovery. The initial aphasia score was the strongest predictor of AOS recovery.

**Conclusion:** Our findings confirm the common occurrence of AOS and aphasia in left hemisphere stroke patients with a hand motor impairment. Recovery was similar across speech-language and motor domains, even in patients with severe impairment, supporting the shared recovery hypothesis and that similar brain recovery mechanisms are involved in speech-language and motor recovery post stroke. These observations contribute to the knowledge of AOS and its relation to motor and language functions and add information that may serve as a basis for future studies of post stroke recovery. Studies including neuroimaging and/or biological assays are required to gain further knowledge on shared brain recovery mechanisms.

## Introduction

Stroke patients often suffer from multiple impairments. A left middle cerebral artery (MCA) stroke with damage to the left inferior frontal cortex and precentral cortex often causes a right-sided hemiparesis, non-fluent (Broca's) aphasia and may also lead to apraxia of speech (AOS) (1, 2). AOS is traditionally described as an impairment that occurs at an intermediate level of speech production; it is neither regarded as a linguistic impairment (aphasia), nor as a problem with speech motor execution (dysarthria). Instead, it is defined as a deficit in planning and programming speech motor movements (3). It has been argued that the impairments in AOS reflect a disconnection between linguistic and motor processes (4), and suggested that the association between AOS and right-sided motor impairments is stronger than that between aphasia and such motor impairments (2). The core symptoms associated with AOS include a slow rate of speech, disturbed prosody and impaired articulation with sound errors that are predominately distortions (5). Non-verbal oral apraxia (NVOA), an impairment of non-speech volitional movements, is frequent especially in severe AOS (2). AOS after stroke often co-occurs with aphasia (6, 7) and patients with non-fluent aphasia often also have AOS (2). However, quantitative information on the prevalence of AOS is limited. At the Mayo Clinic Speech Pathology practice, AOS was documented as the primary communication disorder in 4.7% of the patients with motor speech disorders. The prevalence would presumably be much higher if co-occurring AOS was included in the data (2). Although there is a consensus that speech-language functions are supported by predominantly left lateralized brain regions, there is also an ongoing debate about the contribution of the right hemisphere (RH) in speech production. Several neuroimaging studies have examined the RH's involvement and proposed a bilateral organization of the speech sensory–motor system (8, 9).

Numerous studies have described a close connection between hand motor behavior and speech-language functions. Corballis (10) claimed that language evolved from manual gestures, and that this transition can be traced by studying changes in the function of Broca's area. Several studies have reported a significant activation of Brodmann area 44 in the posterior portion of Broca's area during manual action, e.g., Binkofski et al. (11) and Gerardin et al. (12). In post stroke patients, Meister et al. (13) reported enhanced excitability in the non-dominant hand motor area during reading. This effect had earlier been found only in the language-dominant hemisphere in healthy individuals (14). Harnish et al. (15) studied five chronic post-stroke patients with upper extremity hemiparesis and aphasia during the course of 6 weeks of high intensive motor therapy. All exhibited both hand motor and language improvements, in the three patients with the largest motor improvements also significant language improvements were observed. Meinzer et al. (16) reported that transcranial direct current stimulation over the left primary motor cortex induced long lasting language improvements. Both naming ability and functional communication were ameliorated in individuals with chronic post-stroke aphasia, adding support for a close connection between speech-language and motor systems.

There is a shortage of research involving patients with AOS in an acute/subacute phase after stroke and of studies that investigate how AOS symptoms evolves over time (17, 18). Studies of speech-language recovery after stroke have mainly focused on aphasia [e.g., see (19, 20)] and very limited information exists about the dynamics of recovery from AOS relative to concomitant aphasia. In a single case study, Mauszycki et al. (21) found a parallel recovery pattern of AOS and aphasia over the first 8 months after stroke. Baumgaertner et al. (22) described a disparate recovery process during the first 4 weeks in a single post stroke patient that showed improvement from aphasia but not from AOS. Primaßin et al. (23) examined possible interactions of motor and speech-language processes after 7 weeks of intensive language and motor therapy in four patients in a subacute or chronic phase after stroke. In this study sample, dissociation in the recovery of AOS vs. aphasia was noticed with a tendency of more persistent AOS symptoms compared to those related to aphasia.

Most spontaneous language and motor recovery is assumed to occur within the first 3 months after stroke (24), supporting the hypothesis of common spontaneous recovery coherent with general plasticity mechanisms (25). However, studies have shown that the extent and timing of recovery at the inter-individual level vary considerably, both regarding hand motor (26) and speech-language recovery (27, 28). Traditionally, motor recovery is regarded to start earlier than language recovery in post stroke patients (1), and studies have reported that cognitive functions continue to recover after 3 months, for example language (29) and visual attention (30). There are also alternative hypotheses, referred to in e.g., Primaßin et al. (23), about competitive vs. additive interactions in the recovery process. Competitive interactions would entail that during the course of a general recovery, one domain would draw a proportionately greater amount of neural resources at the disadvantage of a “competing” domain. The alternative assumption is that there is a positive interaction, and that recovery in one domain would benefit also to the recovery in other domains. Recovery has yet most often been studied in a single domain within selected patient groups and few studies address both motor and speech-language abilities (31). Knowledge about determinants of concurrent recovery is therefore missing (23). This is particularly the case for patients with severe speech-language impairments, which often are excluded from studies (32). As a consequence, the generalization of research findings relating to aphasia and AOS patients is limited (33). The exclusion of patients with a severe speech-language impairment also create an imbalance in the recruitment of patients with a hemiparesis in the dominant hand (34).

Among the predictors for post stroke recovery, initial severity, lesion site and lesion size are often proposed as important factors (35). The relation between lesion volume and recovery has been addressed in several studies. Generally, large left hemisphere lesions are associated with poor language recovery whereas smaller lesions are assumed to have a better prognosis (36, 37). Trupe et al. (38) found that patients with persistent AOS had large infarcts involving most of the territory supplied by the superior division of the left MCA. However, research has questioned the role of global lesion size as a reliable correlate for motor and language recovery and its importance as a prognostic factor has been debated (39, 40).

Since basic knowledge on recovery from AOS and its relation to motor and language functions is missing, we set out to describe recovery occurring across domains. A prospective longitudinal observational study design was applied. Given previous findings supporting similar recovery across domains (1) we set out to test the hypothesis that motor and speech and language recovery would be similar in stroke patients presenting with initial arm paresis.

The aims of the study were threefold:

1. To investigate the prevalence of AOS and aphasia in patients with arm and hand motor impairment in an early phase after first ever stroke. Since studies point to shared neurophysiological mechanisms (2), we hypothesized that the prevalence of both AOS and aphasia would be high in the patient group with LH lesions.
2. To describe recovery patterns of AOS, aphasia and hand motor impairment spanning all severity levels, from a subacute phase at 4 weeks to a follow-up at 6 months. Given initial accounts of similar recovery of aphasia and motor impairment (15), we hypothesized that AOS and aphasia recovery would be closely related to hand motor recovery, even in patients with severe initial impairment, reflecting shared plasticity mechanisms.
3. To explore factors predicting recovery from AOS. In line with current findings (27, 40), we also predicted that AOS and aphasia recovery would not be related to lesion volume.

## Methods

### Participants

**Figure 1:**
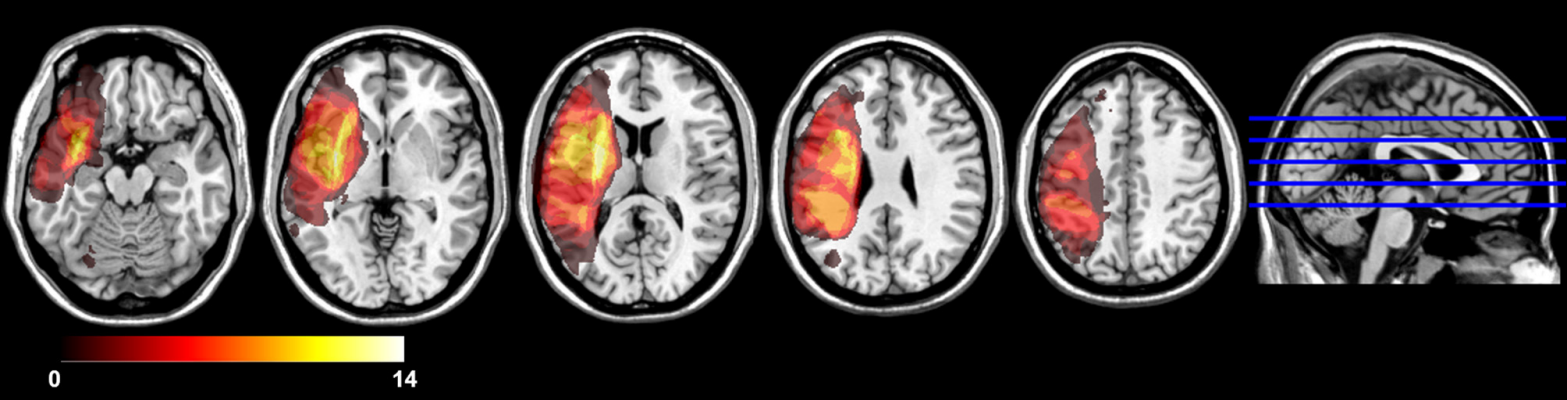
Lesion overlap map (*n* = 14) (1 missing data). All lesions were in LH MCA territory. Lesion overlap was greatest in subcortical white matter in striatocapsular region.

The study was performed as a part of the ProHand study, a prospective longitudinal study designed to identify key determinants for recovery of hand motor function after a first stroke (ClinicalTrials.gov Identifier: NCT02878304). It was approved by the Regional Ethical Review Board in Stockholm and all participants provided informed consent prior to participation. Since the study included patients with severe aphasia, both oral and written information were modified and presented in an aphasia friendly manner to make it accessible also to individuals with compromised language skills. *Inclusion criteria:* (1) Patients aged ≥18 years admitted to inpatient care after first ever-stroke (2) clinical evidence of hand motor deficits based on neurological examination and medical records (3) time of enrolment: between 2 and 6 weeks after stroke onset (4) Swedish as first language, (5) awake, alert and capable of participating in assessment procedures. *Exclusion criteria:* (1) Incapability to understand and comply with instructions (for patients with aphasia presented in an adapted format) (2) Other disorders that may affect hand function (e.g., other neurological conditions, arthritis), (3) Cerebellar lesions (4) Report of claustrophobia or metal object in body (5) Presence of other neurological, psychiatric or medical conditions that preclude active participation. Behavioral assessments and brain imaging examinations were conducted at two time points; the first (A1) at 2–6 weeks after stroke onset and the follow-up (A2) at 6 months. Seventy participants were assessed at A1. Half of the group (*n* = 35) suffered from a right hemisphere (RH) lesion, the other half a left hemisphere lesion (LH) (for demographics and clinical details see Table 1). Complete follow-up assessments at A2 were performed in 15 participants with speech-language impairment at A1. This subgroup all had LH lesions. As can be seen in Table 2 and Figure 1, all in this group had middle cerebral artery lesions. Most were caused by ischemic strokes, but three participants had haemorrhagic strokes. In one case, the lesion was subcortical only; all other participants had cortical lesions extending into the subcortical white matter. The mean lesion volume was 133.8 cm3.

**Table 1:** Participant characteristics at A1, LH lesioned (*n* = 35).

**Variables**	**Measures**		**All** ** (*n* = 35)**	**No SLI** ** (*n* = 10)**	**Aphasia** ** (*n* = 5)**	**Aphasia and AOS** ** (*n* = 20)**	**Group difference** **(sig.)**
Age	Mean (SD) Median		53.3 (8.4)54	57.7 (4.7)58	51.0 (4.9)53	51.6 (9.8)53	0.134^a^
Sex	N (%)	Females	6 (17)	2 (20)	0 (0)	4 (20)	
		Males	29 (83)	8 (80)	5 (100)	16 (80)	
Lesion Volume cm3	Mean (SD) Median		94.9 (102.8)45.5	49.4 (104.3) 4.7	88.6 (111.6) 25.6	120.9 (96.8) 117.1	0.051^a^
Stroke type	N (%)	Ischemic	24 (69)	7 (70)	4 (80)	13 (65)	
		Hemorrhage	11 (31)	3 (30)	1 (20)	7 (35)	
FM-UE total (60p)	Mean (SD) Median		25.1 (24.9)16	39.7 (22.3) 51.5	37.6 (27.0) 57.0	14.7 (21.5)2.0	0.020^a^^*^

*SLI, speech-language impairment, Aphasia diagnoses according to A-NING test score, AOS diagnoses according to ASRS, Percent is calculated as proportion within the respective group*.

a *Kruskal Wallis Test*,

* *Significant at p < 0.05, Bonferroni corrected pairwise comparison*.

*AOS + Aphasia compared no SLI p = 0.04, Aphasia only compared to AOS + Aphasia p = 0.17, Aphasia only compared to no SLI p = 1.0*.

**Table 2:** Participant characteristics and lesion descriptives (*n* = 15).

**ID**	**Sex**	**Age**	**Stroke**	**Les. vol.cm3**	**Lesion location Vascular territory, involved region**
1	F	52	I	30.6	MCA, C and SC
2	M	65	I	121.9	MCA, C and SC
3	M	57	I	32.9	MCA, C and SC
4	F	39	I	117.1	MCA, C and SC
5	M	31	H	167.7	MCA, C and SC
6	M	50	I	115.1	MCA, C and SC
7	M	45	H	143.2	MCA, C and SC
8	M	39	I	175.6	MCA, C and SC
9	F	62	I	^*^	MCA, SC
10	M	53	I	270.2	MCA, C and SC
11	M	61	I	160.2	MCA, C and SC
12	M	44	I	122.7	MCA, C and SC
13	M	54	H	25.6	MCA, C and SC
14	M	54	I	73.1	MCA, C and SC
15	M	48	I	317.8	MCA, C and SC
Mean(SD)		50.3 (9.4)		133.8 (84.7)	
Median		52		122.3	

*F, female; M, male; I, ischemic stroke; H, hemorrhagic stroke; MCA, middle cerebral artery; C, cortical; SC, subcortical*.

* *Data missing*.

### Behavioral Assessments

Presence and degree of AOS was examined using the Apraxia of Speech Rating Scale 2.0 (ASRS 2.0) (41, 42). The ASRS has been applied in several studies and was recently found as a reliable indicator of AOS after stroke (43). Total maximum score is 52, higher scores reflect greater severity, cut-off value for an AOS diagnosis ≥8 points. To assess presence, degree and type of aphasia, the Neurolinguistic Aphasia Examination (A-NING) was used (44). A-NING is a standardized test and includes evaluation of seven linguistic modalities: “oral expression abilities,” “repetition,” “auditory comprehension,” “reading comprehension,” “reading aloud,” “dictation,” and :informative writing.” The maximum result is 220 points, with a corresponding aphasia index of 5.0. The cut-off value for an aphasia diagnosis is 208 < (index 4.8) (45). For visual confrontation naming ability, the Boston Naming Test (BNT) was applied (46). The BNT is a frequently used assessment instrument internationally, both in research and clinical settings (47). Maximum result is 60 and the cut-off value ≤ 47–55 based on Swedish-language norms (48). For assessment of non-verbal oral apraxia (NVOA), a screening instrument developed by Josephs et al. (49) and Botha et al. (50) was applied. This 8-item protocol consists of four gestures repeated twice. Total maximum score is 32, whereas the recommended cut-off for a NVOA diagnosis is <29. To assess hand motor function, the motor domain of the Fugl-Meyer assessment for the upper extremity (FM-UE) was applied (51). The FM-UE is a standard outcome measure in clinical stroke research and has shown excellent inter- and intrajudge reliability and construct validity (52, 53). With the three reflex items excluded, maximum total score is 60 (54). Upper extremity motor impairment level was classified as severe impairment (FM-UE <19), moderate (FM-UE 19–47), and mild (FM-UE >47) (55).

### Magnetic Resonance Imaging

For description of lesion size and location, brain imaging was performed with an Ingenia 3.0T MR system (www.usa.philips.com) with an 8HR head coil. High-resolution T1-weighted anatomical images were acquired using TFE 3D (3-dimensional gradient echo-based sequence): field of view, 250 × 250 × 181 mm; matrix, 228 × 227; slice thickness, 1.2 mm; slice spacing, 0.6 mm; and number of slices, 301 (echo time, shortest; relaxation time, shortest). T2 fluid attenuated inversion recovery images were also acquired. Anatomical T1-images were normalized to Montreal Neurological Institute template using SPM12 (www.fil.ion.ucl.ac.uk/spm/software/spm12/). Cost function masking was used to avoid distortion of lesion by normalization procedure, and the images were inspected visually to ensure adequate normalization. Lesion maps were manually drawn on all axial slices of native space T1 weighted anatomical images using MRIcron (https://people.cas.sc.edu/rorden/mricron/index.html) by researcher (JP) and verified by an experienced neurologist, who was blinded to all clinical data except the lesioned hemisphere.

### Statistical Methods

The amount of recovery was defined as the percentage that a participant improved over time on a test in relation to the possible maximum improvement on that specific measure. Recovery ratio was calculated as the absolute amount of recovery (change from the subacute score, A2–A1) divided by the amount corresponding to full recovery (difference between subacute score and maximum score, max—A1). To minimize known ceiling effects, patients with results at ceiling at the initial assessment were excluded from the analysis (56). To test the strength of association between recovery ratios in different domains and between behavioral score result at A1 and A2, Spearman's correlation coefficients were calculated. The non-parametric option was chosen to avoid a strong influence of possible outliers in the relatively small dataset. Between-group differences were tested using Kruskal-Wallis Test. For within-group differences, Related-Samples Wilcoxon Signed Rank Test was applied. To investigate predictors of AOS recovery, univariate regression analyses were performed. Since this data was not severely skewed, the parametric option could be allowed in order to explore the explained variance among the predictors. Statistical significance level was set at *p* < 0.05. A separate subgroup analysis was undertaken in participants with severe speech-language impairments at A1, since descriptions of recovery in these patients are scarce.

## Results

### Prevalence of AOS and Aphasia at 4 Weeks After Stroke Onset

All participants with a speech-language impairment had LH lesions (*n* = 35). In this group, 20 participants (57%) had AOS with concomitant aphasia. Five participants (14%) had aphasia without concomitant AOS, while 10 participants (28.6%) showed no signs of AOS or aphasia. Unilateral upper motor neuron dysarthria was present in approximately half of the group with AOS and/or aphasia, but all to a mild degree that did not interfere the speech-language measurements. As seen in Table 1, a Kruskal-Wallis test showed a statistically significant difference in FM-UE score across the three participant groups (*H* = 7.8, *p* = 0.02), with a much lower median FM-UE score in the group with AOS and aphasia than in the groups with aphasia only or with no speech-language impairment. Pairwise *post-hoc* comparisons indicated that the only significant difference was between the group with AOS and concomitant aphasia and the group without speech-language impairment (*p* = 0.04).

### Recovery in Speech-Language and Hand Motor Domains

Recovery was studied in 15 of the 25 LH lesioned participants presented in Table 1 with a speech-language impairment. The missing data was due to the initial design of the ProHand study, where a follow-up assessment of speech-language functions initially was not included. The second assessment occasion was instead added to the protocol 18 months after the data collection had started.

In this group (Table 3), 12 also had AOS at A1. The mean ASRS was 22.8, indicating moderate/severe AOS. Aphasia severity ranged from mild to very severe, the mean A-NING value was 93.6 (A-NING aphasia index 2.0) which corresponds to severe aphasia. The BNT scores were generally low and correlated significantly with A-NING scores. All participants with AOS had concomitant NVOA with significantly correlated severity. The initial ASRS results were also significantly correlated with results from A-NING and BNT. The mean FM-UE score was 14.9 with a median value of 2 points reflecting an almost total arm and hand paresis. FM-UE score correlated significantly with measures of NVOA but did not reach significance in relation to scores of ASRS, A-NING and BNT. No correlation was found between lesion volume and behavioral assessments (Table 4).

**Table 3:** Behavioral measurements, total score results, assessment 1 (A1) and 2 (A2) (*n* = 15).

**ID**		**ASRS**		**A-NING**		**FM-UE**		**BNT**		**NVOA**	
		**A1**	**A2**	**A1**	**A2**	**A1**	**A2**	**A1**	**A2**	**A1**	**A2**
1		44	35	35	95	0	4	0	21	16	17
2		18	15	36	46	0	2	1	2	6	7
3		23	15	26	86	1	9	0	5	2	9
4		22	14	70	120	2	10	12	38	17	20
5		17	9	134	202	2	29	49	51	18	30
6		17	5	160	200	1	7	15	48	21	32
7		29	28	22	25	0	2	0	0	0	2
8		28	18	38	173	16	34	0	44	5	21
9		10	2	207	216	2	23	45	50	27	32
10		6	2	199	218	57	60	57	57	29	32
11		9	2	150	190	21	51	17	43	12	20
12		7	7	118	192	7	40	32	42	26	27
13		7	3	181	206	57	60	52	52	23	32
14		32	23	18	133	58	60	0	49	26	30
15		25	24	10	25	0	4	0	0	0	0
	Mean	19.6	13.5	93.6	141.8	14.9	26.3	18.7	33.5	15.2	20.7
	(SD)	10.9	10.5	72.8	71.0	22.8	23.0	22.1	21.4	10.4	11.5
	Median	18	14	70	173	2	23	12	43	17	21

*ASRS, Apraxia of speech rating scale, max 52, cut-off value for AOS ≥ 8 points; A-NING, A-ning neurolinguistic aphasia examination, total score results and severity index classification: very severe/Global ≤ 42 (INDEX ≤ 0.9), Severe ≤ 82 (INDEX ≤ 1.8), Moderate ≤ 170 (INDEX ≤ 3,8), Moderate/mild ≤ 192 (INDEX ≤ 4.4), Mild ≤ 208 (INDEX ≤ 4.7), No Aphasia > 208 (INDEX > 4.8), maximum 220 points; FM-UE, upper extremity motor portion of the Fugl-Meyer scale, max 60, cut-off level severe—moderate imp. 19 ± 2 points, moderate—mild 47 ± 2 points; BNT, Boston naming test, max 60, cut-off value ≤ 47–55 based on normative data (education); NVOA, non verbal oral apraxia protocol, max 32, cut-off value for NVOA diagnosis < 29 points*.

**Table 4:** Correlation between assessments at A1 and A2 (*n* = 15).

**Measurements**		**Spearman's rho**	**Sig**.
**Assessment 1**			
ASRS vs.	A-NING	−0.83^**^	<0.001
	FM-UE	−0.46	0.08
	NVOA	−0.58^*^	0.02
	BNT	−0.92^**^	<0.001
	Lesion volume	−0.18	0.55
FM-UE vs.	A-NING	0.45	0.10
	NVOA	0.64^*^	0.01
	BNT	0.48	0.07
	Lesion volume	−0.01	0.96
A-NING vs.	BNT	0.89^**^	<0.001
	NVOA	0.68^**^	0.005
	Lesion volume	0.02	0.95
NVOA vs.	BNT	0.72^**^	0.003
	Lesion volume	−0.21	0.48
BNT vs.	Lesion volume	0.11	0.71
**Assessment 2**			
ASRS vs.	A-NING	−0.83^**^	<0.001
	FM-UE	−0.57^*^	0.03
	NVOA	−0.71^**^	0.003
	BNT	−0.68^**^	0.005
	Lesion volume	−0.09	0.76
FM-UE vs.	A-NING	0.71^**^	0.003
	NVOA	0.69^**^	0.004
	BNT	0.80^**^	<0.001
	Lesion volume	−0.01	0.97
A-NING vs.	BNT	0.94^**^	<0.001
	NVOA	0.94^**^	<0.001
	Lesion volume	0.01	0.98
NVOA vs.	BNT	0.94^**^	<0.001
	Lesion volume	−0.16	0.59
BNT vs.	Lesion volume	−0.04	0.90

* *Significant at p < 0.05*,

** *Significant at p < 0.01*.

At A2, total results of ASRS, A-NING and FM-UE, respectively demonstrated statistically significant improvements (Related-Samples Wilcoxon Signed Rank Test; ASRS *p* < 0.002, A-NING and FM-UE *p* < 0.001). Two of the former 15 participants with aphasia showed a complete recovery while 3 still had severe to very severe aphasia. Out of the 12 participants with AOS at A1, 9 still had AOS of varying severity. The ASRS scores correlated significantly with A-NING, BNT, and NVOA results. Three participants showed a complete hand motor recovery with maximum results on the FM-UE, whereas several participants still had a severe hand motor impairment. At this timepoint, the FM-UE total score also correlated significantly with all speech-language results (Table 4).

### Comparison of Recovery Across Domains

**Figure 2:**
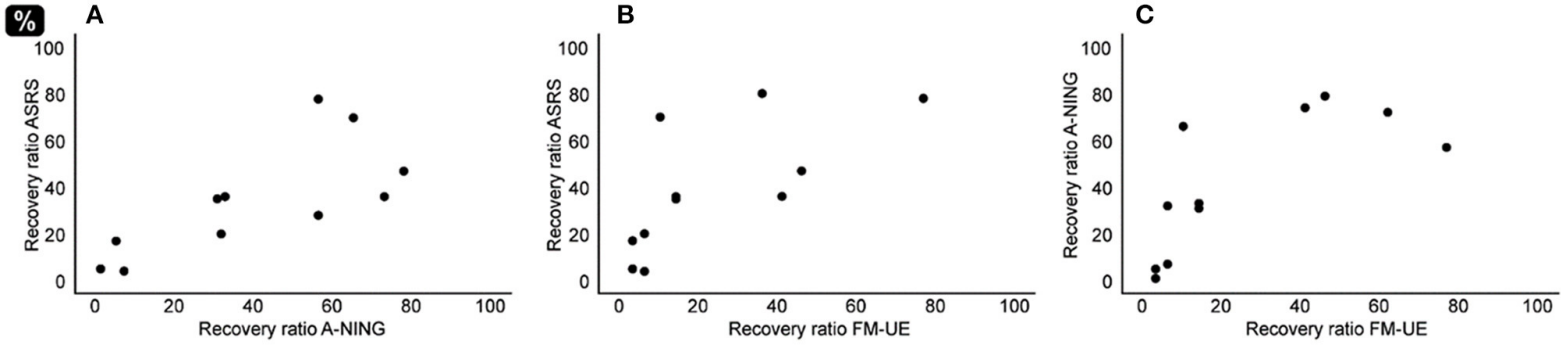
Relation recovery ratios. **(A)** AOS recovery ratio (ASRS) correlated with recovery ratio of aphasia (A-NING) (Rho = 0.80, *p* < 0.01). **(B)** AOS recovery ratio (ASRS) correlated with recovery ratio of hand motor impairment (FM-UE) (Rho = 0.78, *p* < 0.01). **(C)** Aphasia recovery (A-NING) correlated with recovery of hand motor impairment (FM-UE) (Rho = 0.80, *p* < 0.01).

At a group level, the mean recovery ratios varied between 26% for FM-UE up to 48% for A-NING; for ASRS the mean recovery was 38% (Table 5). At an inter-individual level, the magnitude of the recovery varied widely in all of the applied behavioral measurements. In analyses of the relation between recovery ratios, a parallel pattern with significant correlations between hand motor and speech-language domains was found (Figure 2 and Table 6). In all domains, no significant correlations were found between lesion volume and recovery ratio (Table 6).

**Table 5:** Descriptive statistics, recovery ratios.

	**A-NING REC**.	**ASRS REC**.	**BNT REC**.	**NVOA REC**.	**FM-UE REC**.
	**(%) (*n* = 14)**	**(%) (*n* = 12)**	**(%) (*n* = 12)**	**(%) (*n* = 14)**	**(%) (*n* = 12)**
Mean (SD)	48 (29.4)	38 (26.3)	38 (30.5)	45 (39.3)	26 (25.2)
Median	57	35	35	31	14
Min.	1	4	0	0	3
Max.	90	80	82	100	77

*Total score results at ceiling at A1 excluded in the analyses; A-NING > 200; ASRS < 9; BNT 2 points below the normative value, NVOA ≥ 28; FM-UE ≥ 57*.

**Table 6:** Relation between recovery ratios.

	**Measurements**	**Spearman's rho**	**Sig**.
ASRS vs.	A-NING	0.80^**^	0.003
	FM-UE	0.78^**^	0.004
	NVOA	0.84^**^	0.001
	BNT	0.55	0.055
	Lesion volume	−0.005	0.989
FM-UE vs.	A-NING	0.80^**^	0.003
	NVOA	0.59^*^	0.045
	BNT	0.65^*^	0.029
	Lesion volume	0.25	0.464
A-NING vs.	BNT	0.84^**^	0.001
	NVOA	0.74^**^	0.004
	Lesion volume	0.25	0.391
NVOA vs.	BNT	0.72^**^	0.008
	Lesion volume	−0.28	0.357
BNT vs.	Lesion volume	−0.20	0.563

* *Significant at p < 0.05*,

** *Significant at p < 0.01*.

### Prediction of AOS Recovery

Univariate linear regression analysis revealed initial A-NING total score as the strongest predictor for the magnitude of AOS recovery ratio; 84% of the variance could be explained by this factor. The initial ASRS score accounted for 52% of the variance, initial BNT score for 45%, initial NVOA for 35%, while the initial FM-UE score had no explanatory power (0.6%). The regression coefficients for lesion volume and age were also low and not statistically significant (Table 7).

**Table 7:** Univariate linear regression analyses (*n* = 15).

**Outcome**	**Predictors**	**Explained variance** **/R square**	***p*-value**
ASRS recovery ratio	A-NING total at A1	0.836^**^	<0.0001
	ASRS total at A1	0.525^**^	0.008
	BNT total at A1	0.448^*^	0.017
	NVOA total score at A1	0.353^*^	0.04
	Age	0.047	0.497
	FM-UE at A1	0.006	0.818
	Lesion volume cm3	0.001	0.942

* *Significant at p < 0.05*,

** *Significant at p < 0.01*.

### Recovery in Speech-Language and Hand Motor Domains in the Group With Severe Aphasia

Separate analyses were performed in a subgroup of 7 participants (ID 1, 2, 3, 7, 8, 14, 15 in Table 3) with very severe aphasia at the initial assessment (A-NING severity index ≤0.9). At A1, all in this group had AOS, the mean ASRS total score was 28.4 indicating severe AOS. The majority also had severe NVOA, only one participant (ID 14) showed a moderate impairment. Almost no naming ability was demonstrated; one participant scored 1/60 points on BNT, the others 0/60. Except for two participants (ID 8 and 14), all in this subgroup had an almost total arm and hand paresis (FM-UE 0 or 1 point). Participant ID 8 had a FM-UE of 16, indicating severe motor impairment, ID 14 had a very discrete hand motor impairment (FM-UE = 58). The lesion volume varied from 30.7 cm3 to maximum 317.8 cm3, with a mean value of 127.9 cm3.

**Figure 3:**
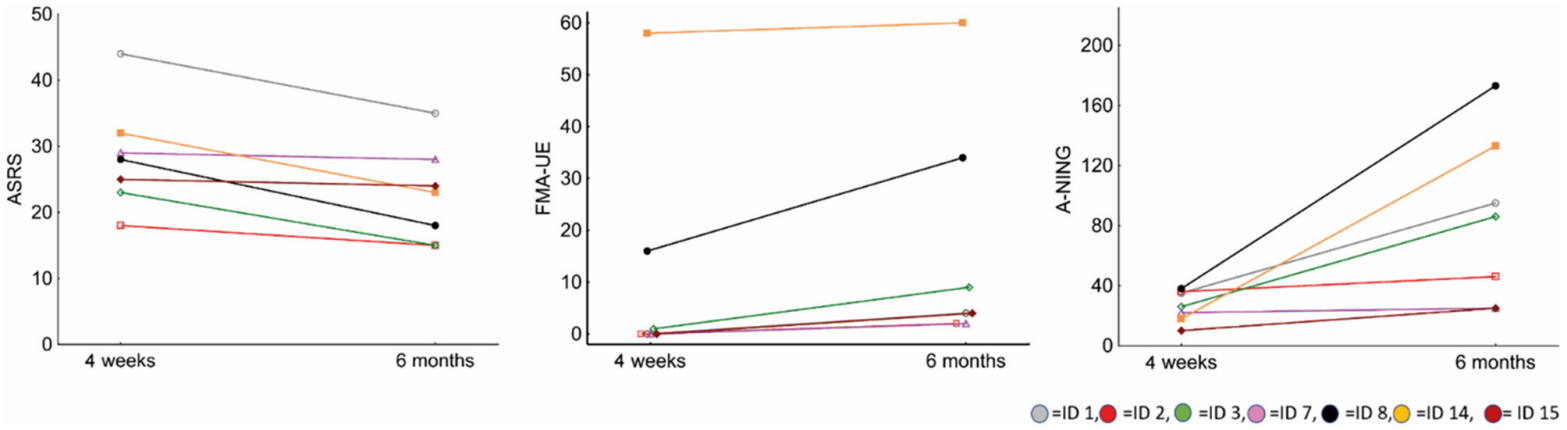
Total score changes between A1 and A2 in ASRS, FM-UE, and A-NING. In ASRS, higher scores reflect greater severity.

At A2, four participants showed aphasia recovery with A-NING scores in the moderate impairment range (A-NING severity index ≤1.9–3.8). One participant progressed from very severe to severe aphasia (A-NING severity index ≤1.0) while two participants still had very severe (global) aphasia. The same pattern was found in naming ability and the results of BNT and A-NING showed a strong correlation (rho = 0.96). All 4 participants that showed a clinically significant improvement in aphasia (i.e., moved ≥1 index in A-NING) also improved a minimum of 5 points on the ASRS, the remaining 3 participants showed very limited/no recovery. In hand motor function, the overall FM-UE result remained low (mean 16.4, median 4.0). The participant with very discrete impairment at A1 showed a complete recovery (from 58 to 60 points). Only one participant, ID 8, showed a clinically significant hand motor improvement (i.e., recovery ≥9 points in FM-UE) (57) (Figure 3).

### Comparison of Recovery Across Domains in the Group With Severe Aphasia

**Figure 4:**
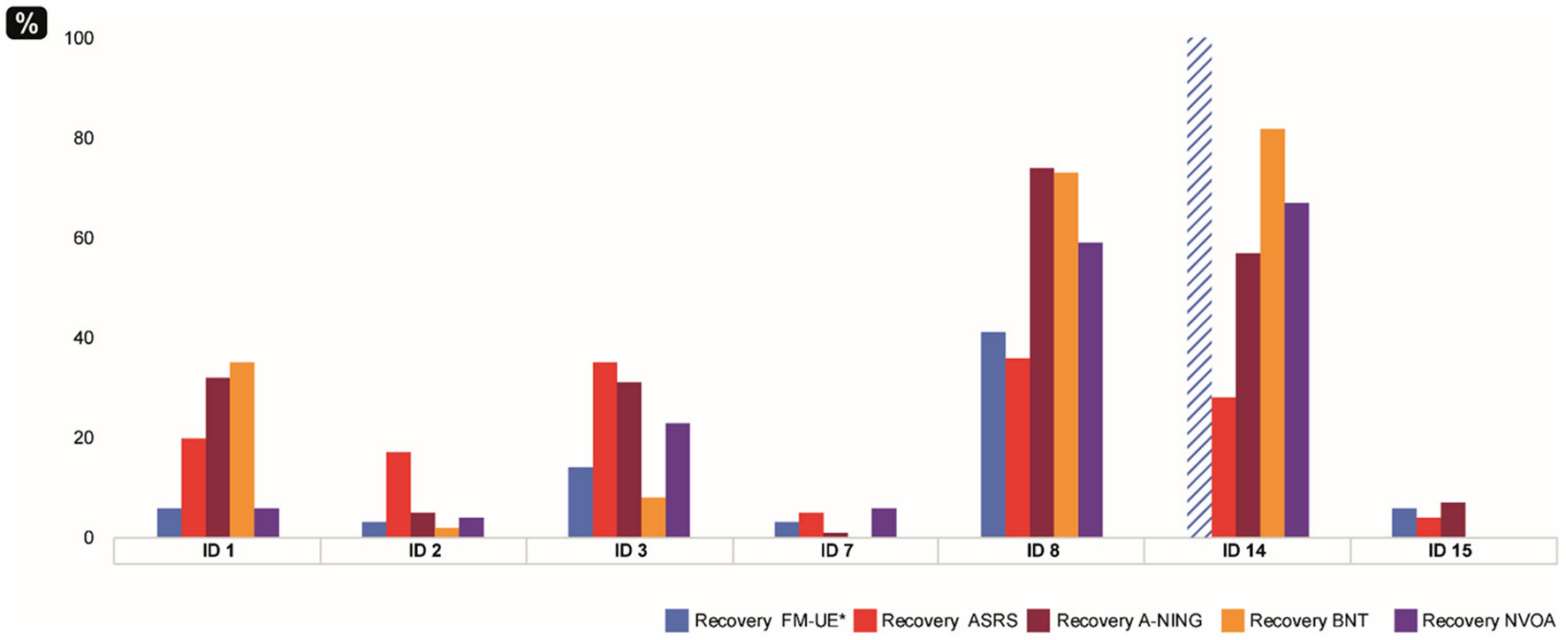
Recovery ratios in participants with severe initial aphasia (*n* = 7). *Blue striped plot shows FM-UE result at ceiling at A1.

Low recovery ratios were found in all three domains; the mean value was 30% for A-NING, 23% for ASRS and 12% for FM-UE. Higher recovery magnitudes were found in only two participants, ID 8 and ID 14, with recovery ratios around 70% in the language domain (Figure 4). A parallel pattern across language and motor domains was noticed also in this subgroup. There were significant correlations between AOS and aphasia recovery (ASRS vs. A-NING rho = 0.79^*^) and between hand motor and aphasia recovery (FM-UE vs. A-NING rho = 88^*^), while the correlation between AOS recovery and hand motor recovery did not reach significance (ASRS vs. FM-UE rho= 0.74). The lesion volume did not correlate significantly with the recovery ratio in any of the domains (Table 8).

**Table 8:** Relation recovery ratios in the group with severe aphasia (*n* = 7).

**Measurements**		**Spearman's rho**	**Sig**.
ASRS vs.	A-NING	0.79^*^	0.04
	FM-UE	0.74	0.09
	NVOA	0.83^*^	0.02
	BNT	0.81^*^	0.03
	Lesion vol. cm3	−0.36	0.43
FM-UE vs.	A-NING	0.88^*^	0.02
	NVOA	0.69	0.13
	BNT	0.72	0.11
	Lesion vol. cm3	0.09	0.87
A-NING vs.	BNT	0.90^**^	0.006
	NVOA	0.72	0.07
	Lesion vol. cm3	−0.18	0.70
NVOA vs.	BNT	0.84^*^	0.02
	Lesion vol. cm3	−0.32	0.48
BNT vs.	Lesion vol. cm3	−0.43	0.33

* *Significant at p < 0.05*,

** *Significant at p < 0.01*.

## Discussion

The findings of this study provide original data on the prevalence of AOS and aphasia in post stroke patients with arm and hand motor impairment. In addition, a parallel recovery pattern for AOS, aphasia and hand motor domains across all severity levels is demonstrated. These observations add to the knowledge of AOS and its relation to motor and language functions, and provide information that may serve as a basis for future studies of post stroke recovery mechanisms.

### Prevalence of AOS and Aphasia in Patients With Arm and Hand Motor Impairment

We examined 70 patients with a hand motor impairment after first ever stroke with a comprehensive speech-language assessment battery. Since the RH involvement in the speech-motor network has been discussed, most often considered to be involved in feed-back control and/or in lower levels of speech production (58, 59), the initial study sample also included 35 patients with RH lesions. However, no participant with AOS or aphasia after a RH lesion was found. Instead, the majority (71%) of the participants with a left hemisphere lesion had aphasia. Over half of the group (57%) also suffered from AOS, several with moderate to severe impairments. The co-occurrence of AOS and aphasia was high; all participants with AOS had aphasia and 80% of all participants with aphasia also had AOS. These findings support the current view regarding the common occurrence of AOS with concomitant aphasia after a LH stroke (2) and add new quantitative information to the limited data regarding the prevalence of AOS in early post stroke patients.

### Similar Speech-Language and Motor Recovery Post-stroke

In accordance with our hypothesis, a parallel recovery pattern was found between speech-language and hand motor domains. This pattern was also apparent in severely impaired patients. Neither recovery ratio nor severity of impairment co-varied with lesion volume, coherent with recent reports on hand motor recovery (60).

In speech-language domains, strong correlations were found both between the level of impairment in all measurements of behavioral functions and of the magnitude of recovery during the first 6 months. Consistent with earlier studies, e.g., Dronkers et al. (61) and New et al. (62), a frequent co-occurrence of NVOA and AOS was found. The severity of AOS and NVOA was significantly correlated, which also is in accordance with findings by Botha et al. (50) in studies of patients with primary progressive aphasia and/or primary progressive AOS. In contrast to earlier findings of a disparate AOS and aphasia recovery (22, 23), a parallel pattern was noticed between recovery from AOS and aphasia. The covariance was more apparent between the recovery ratio of AOS and the recovery ratios from the broad aphasia score of A-NING, covering all language modalities, compared to recovery in naming ability measured by BNT that did not reach statistical significance. A correlated parallel pattern was also apparent for NVOA recovery ratio in relation to recovery from both AOS and aphasia.

Significantly correlated recovery ratios were also found between hand motor function compared to recovery of speech and language functions. This parallel pattern was also observed and confirmed in the pairwise correlation analyses between behavioral total score results, with a tendency of becoming even more strongly correlated at A2 (Table 4). Even though several earlier studies have suggested a link between motor and language abilities [e.g., see (15, 16, 23, 63)] studies focusing on simultaneous motor and language impairments after stroke and on concurrent recovery are scarce. To our knowledge, the only study so far that investigated longitudinal recovery after stroke in multiple domains is Ramsey et al. (1), who reported similar patterns of recovery across motor and cognitive domains indicative of common underlying mechanisms. Beside the impact of anatomical proximity and changes in important white matter tracts to the recovery process, the authors also reported correlated recovery patterns in specific clusters of behavioral deficits with the underlying lesion areas distributed in distant cortical regions, often in different vascular territories (1). The parallel recovery pattern that was found in our behavioral hand motor and speech-language data extends these findings, indicating that shared plasticity mechanisms driving recovery across language and motor domains also apply to AOS recovery and to recovery in patients with severe speech-language impairments. Our observations do not clarify whether the parallel recovery pattern is specific in regard to these functions; if these findings are due to shared neural correlates for hand motor and speech-language functions or merely reflects anatomical proximity and vascular factors, or may depend upon other general brain-wide plasticity mechanisms in distributed networks. These questions need to be investigated in future studies designed for that purpose. Brain plasticity is operating at different levels, ranging from molecular, cellular and systemic to behavioral/treatment-induced aspects (64) and has been studied both in speech-language domains [for a review, see Kiran and Thompson (37)] and in motor domains [for a review, see Cramer and Riley (65) and Raffin and Hummel (66)]. In speech and language research, several functional and structural neuroimaging studies have shown that both residual and new neural mechanisms can be recruited in the recovery process after stroke [e.g., see (67, 68)]. Genetic (69) and biological (70) factors also likely contribute to recovery across domains. The role of neuroplasticity in speech and language recovery is however not fully understood yet (71).

In our data, there were no signs of a lower aphasia recovery during the first 6 months after stroke in comparison to recovery of hand motor function. Instead, the opposite tendency was seen with several participants showing more improvement in language domains than in hand motor domains. Primaßin et al. (23) stated that there is no prospect of language recovery for stroke patients with no motor improvements. Our results do not unequivocally support this statement. In line with this postulate, the two participants in the very severe aphasia subgroup who reached recovery ratios around 70% in speech-language domains were the only ones in this subgroup without an almost total hand paresis at A1 (ID 8 and 14 in Figure 3). These two divergent participants also improved in hand motor function (ID 14 with initially a very mild impairment showed a complete recover; for ID 8 the recovery ratio for FM-UE was 41%), while the others in this subgroup showed a very discrete hand motor improvement (mean recovery ratio 6%). However, other participants in this study, several with an almost total hand paresis at the initial stage, displayed a limited hand motor recovery but showed a stronger recovery in speech and language domains. The lack of correlation between initial FM-UE score and ASRS recovery also indicates that speech-language recovery is not well-predicted by degree of initial motor impairment (Table 6). These observations do not support the straightforward proposition by Primaßin et al. (23) about the role of the motor cortex in speech and language processes. Instead, our findings show that the role of the motor cortex in speech-language networks still remains unclear and that its specific involvement in speech and language processes needs further examination (16, 72).

It could be argued that although a correlated recovery pattern was found, the differences in the relative amount of change between domains could imply that speech-language recovery exploited resources at the expense of hand motor functions. Admittedly, such an interpretation cannot be totally ruled out. However, the overall pattern with correlated recovery ratios at the individual level, supported by the even more significantly correlated behavioral score result at the follow-up compared to the initial assessment (in Table 4), suggests no signs that there was a competition for resources. It should be acknowledged that beside the shared plasticity mechanisms suggested to be present, other factors undoubtedly also have an impact on the recovery process. As seen in Table 2 and Figure 1, several of the participants suffered from deep subcortical lesions, affecting important white matter tracts. It is known that lesions to specific white fiber tract often results in poorer recovery in different domains, such as the integrity of the arcuate fasciculus for speech-language function (73, 74) and the corticospinal tract for hand motor function (75, 76). The role of white matter damage in relation to shared plasticity mechanisms is beyond the scope of this study and needs to be addressed in studies designed for that purpose with a larger sample of patients.

### Prediction of AOS Recovery

Recovery of AOS was not predictable based on lesion volume, age or the initial FM-UE score result. While the initial impairment level measured by the ASRS could explain about 50% of the variation, the strongest predictor for AOS recovery was the total score at the initial A-NING assessment. This score summarizes language production and comprehension in both spoken and written modalities. Why would such a global measure of language performance predict the recovery of AOS, defined as a motor speech disorder, better than the initial ASRS score? As one possible explanation, our finding could be interpreted by considering contemporary neurocomputational speech production models, e.g., the Gradient Order DIVA (GODIVA) model (77, 78), which includes both feed-forward and feed-back processes. Although AOS traditionally refers to motor planning and programming processes, a clear boundary between linguistic and motor speech processes is not admitted (79). Since speech production is a sensorimotor behavior that depends upon monitoring of input from one or more sensory systems (80), improvement of planning and programming speech motor movements requires that the underlying linguistic and phonological representation of the target word/phrase is intact. This theoretical argument is supported by (clinical) findings that the severity of an accompanying aphasia may hinder successful AOS treatment (2). Early arm and hand motor impairment was not predictive of ASRS recovery (Table 7). As can be seen in Table 3, more than half (60%) of the participants in this study sample had a very severe hand motor impairment with zero or near zero points on the FM-UE at the first assessment. This group showed a substantial variability in ASRS recovery and outcome. Still, when comparing recovery of ASRS and FM-UE at an inter-individual level (Figure 2B), these follow the same recovery pattern; i.e., participants with a limited amount of change in FM-UE also showed a just as limited amount of change in ASRS, and participants with a larger amount of change in FM-UE also recovered accordingly in ASRS.

### Limitations

This study has several limitations. First, it should be noted that the results on prevalence of AOS and aphasia stem from a group of patients with initial upper limb motor impairment. Although the presence of a right sided hemiparesis is considered as very common in post stroke patients with non-fluent aphasia and AOS, our results do not hold for the general stroke population. Secondly, all participants included in the recovery analyses received team-based rehabilitation, with three to five sessions a week of physiotherapy and speech-language therapy during the period. The exact dose and frequency of therapy sessions extent were however not controlled for in this study. However, no participant received any form of specific high intensive therapy, e.g., participated in a period of Intensive Language Action Therapy (81) or in Constraint-Induced Movement Therapy for the upper extremity (82). Thirdly, the recovery in AOS and aphasia was described in a small sub-sample of patients which inevitably limits the statistical strength in the performed analyses. It should also be acknowledged that the sensitivity to change may differ among the applied behavioral assessment instruments. The results should therefore be interpreted with some caution and recovery across speech-language and hand motor domains needs to be further investigated in a larger patient sample. Finally, we acknowledge that our findings do not offer any explanatory answers. Instead, we hope that our observations may serve as a basis for future studies in this area; to identify important prognostic factors and for improvement of targeted treatment interventions for patients with AOS.

## Conclusions

Despite the limited sample size, original longitudinal data was provided including descriptions of both motor and speech/language impairments. Recovery was similar across speech-language and motor domains, even in patients with severe impairment, supporting the shared recovery hypothesis. Future studies including neuroimaging and/or biological assays will permit to gain further knowledge on the shared neural substrates and mechanisms involved in recovery across motor and speech-language domains.

## Data Availability Statement

The raw data supporting the conclusions of this article will be made available by the authors, without undue reservation.

## Ethics Statement

The studies involving human participants were reviewed and approved by Regional Ethical Review Board in Stockholm. The patients/participants provided their written informed consent to participate in this study.

## Author Contributions

Study concept and design: PL, PÖ, ES, CN-D, JP, and HH. Study supervision: PL and PÖ. Data collection: HH and JP. Analysis and interpretation of data: HH, PL, PÖ, JP, ES, MS, and CN-D. Manuscript draft/manuscript writing: HH. Critical revision of manuscript: PL, PÖ, ES, JP, CN-D and MS. All authors have read and approved the submitted version of the manuscript.

## Conflict of Interest

The authors declare that the research was conducted in the absence of any commercial or financial relationships that could be construed as a potential conflict of interest.

## References

[1] Ramsey LE Siegel JS Lang CE Strube M Shulman GL Corbetta M. Behavioural clusters and predictors of performance during recovery from stroke. Nat. Hum. Behav. (2017) 1:0038. 10.1038/s41562-016-0038 28713861 PMC5508212

[2] Duffy JR. Motor Speech Disorders: Substrates, Differential Diagnosis, and Management. NewYork, NY: Elsevier (2020).

[3] Darley FL Brown JR Aronson AE. Motor Speech Disorders. Philadelphia, PA: Saunders (1975). 10.3109/asl2.1975.3.issue-1.03

[4] Laganaro M Croisier M Bagou O Assal F. Progressive apraxia of speech as a window into the study of speech planning processes. Cortex. (2012) 48:963–71. 10.1016/j.cortex.2011.03.010 21513930

[5] McNeil MR. Clinical Management of Sensorimotor Speech Disorders. New York, NY: Thieme (2009).

[6] Graff-Radford J Jones DT Strand EA Rabinstein AA Duffy JR Josephs KA. The neuroanatomy of pure apraxia of speech in stroke. Brain Lang. (2014) 129:43–6. 10.1016/j.bandl.2014.01.004 24556336 PMC4004427

[7] Moser D Basilakos A Fillmore P Fridriksson J. Brain damage associated with apraxia of speech: evidence from case studies. Neurocase. (2016) 22:346–56. 10.1080/13554794.2016.1172645 27264534 PMC6311110

[8] Cogan GB Thesen T Carlson C Doyle W Devinsky O Pesaran B. Sensory-motor transformations for speech occur bilaterally. Nature. (2014) 507:94–8. 10.1038/nature12935 24429520 PMC4000028

[9] Zaca D Corsini F Rozzanigo U Dallabona M Avesani P Annicchiarico L. Whole-brain network connectivity underlying the human speech articulation as emerged integrating direct electric stimulation, resting state fMRI and tractography. Front. Hum. Neurosci. (2018) 12:405. 10.3389/fnhum.2018.00405 30364298 PMC6193478

[10] Corballis MC. From mouth to hand: gesture, speech, and the evolution of right-handedness. Behav. Brain Sci. (2003) 26:199–208. 10.1017/S0140525X03000062 14621511

[11] Binkofski F Buccino G Posse S Seitz RJ Rizzolatti G Freund H. A fronto-parietal circuit for object manipulation in man: evidence from an fMRI-study. Eur. J. Neurosci. (1999) 11:3276–86. 10.1046/j.1460-9568.1999.00753.x 10510191

[12] Gerardin E Sirigu A Lehéricy S Poline JB Gaymard B Marsault C. Partially overlapping neural networks for real and imagined hand movements. Cereb. Cortex. (2000) 10:1093–104. 10.1093/cercor/10.11.1093 11053230

[13] Meister IG Sparing R Foltys H Gebert D Huber W Topper R. Functional connectivity between cortical hand motor and language areas during recovery from aphasia. J. Neurol. Sci. (2006) 247:165–8. 10.1016/j.jns.2006.04.003 16737714

[14] Meister IG Boroojerdi B Foltys H Sparing R Huber W Töpper R. Motor cortex hand area and speech: implications for the development of language. Neuropsychologia. (2003) 41:401–6. 10.1016/S0028-3932(02)00179-3 12559157

[15] Harnish S Meinzer M Trinastic J Fitzgerald D Page S. Language changes coincide with motor and fMRI changes following upper extremity motor therapy for hemiparesis: a brief report. Brain Imaging Behav. (2011) 8:370–7. 10.1007/s11682-011-9139-y 21989635

[16] Meinzer M Darkow R Lindenberg R Floel A. Electrical stimulation of the motor cortex enhances treatment outcome in post-stroke aphasia. Brain. (2016) 139(Pt 4):1152–63. 10.1093/brain/aww002 26912641

[17] Haley KL Shafer JN Harmon TG Jacks A. Recovering with acquired apraxia of speech: the first 2 years. Am. J. Speech Lang. Pathol. (2016) 25:S687–S96. 10.1044/2016_AJSLP-15-0143 27997946

[18] Basilakos A. Contemporary Approaches to the management of post-stroke apraxia of speech. Seminars Speech Lang. (2018) 39:25–36. 10.1055/s-0037-1608853 29359303 PMC5834303

[19] Pedersen PM Vinter K Olsen TS. Aphasia after stroke: type, severity and prognosis. The Copenhagen aphasia study. Cerebrovasc. Dis. (2004) 17:35–43. 10.1159/000073896 14530636

[20] Seghier ML Patel E Prejawa S Ramsden S Selmer A Lim L. The PLORAS database: a data repository for predicting language outcome and recovery after stroke. Neuroimage. (2016) 124(Pt B):1208–12. 10.1016/j.neuroimage.2015.03.083 25882753 PMC4658335

[21] Mauszycki SC Wambaugh JL Wright S. A sub-acute case of resolving acquired apraxia of speech and aphasia. Int. J. Phys. Med. Rehabil. (2014) 2:188–94. 10.4172/2329-9096.1000188

[22] Baumgaertner A Schraknepper V Saur D. Diffrential recovery of aphasia and apraxia of speech in an adolescent after infarction of the left frontal lobe: longitudinal behavioral and fMRI data. Brain Lang. (2005) 95:211–2. 10.1016/j.bandl.2005.07.110

[23] Primaßin A Scholtes N Heim S Huber W Neuschafer M Binkofski F. Determinants of Concurrent motor and language recovery during intensive therapy in chronic stroke patients: four single-case studies. Front. Neurol. (2015) 6:215. 10.3389/fneur.2015.00215 26500606 PMC4598579

[24] Wade DT Wood VA Hewer RL. Recovery after stroke—the first 3 months. J. Neurol. Neurosurg. Psychiatry. (1985) 48:7–13. 10.1136/jnnp.48.1.7 3973623 PMC1028175

[25] Lazar RM Minzer B Antoniello D Festa JR Krakauer JW Marshall RS. Improvement in aphasia scores after stroke is well predicted by initial severity. Stroke. (2010) 41:1485–8. 10.1161/STROKEAHA.109.577338 20538700 PMC2921806

[26] Prabhakaran S Zarahn E Riley C Speizer A Chong JY Lazar RM. Inter-individual variability in the capacity for motor recovery after ischemic stroke. Neurorehabil. Neural Repair. (2008) 22:64–71. 10.1177/1545968307305302 17687024

[27] Lazar RM Speizer AE Festa JR Krakauer JW Marshall RS. Variability in language recovery after first-time stroke. J. Neurol. Neurosurg. Psychiatry. (2008) 79:530–4. 10.1136/jnnp.2007.122457 17846113

[28] Cahana-Amitay D. Redefining Recovery From Aphasia. Oxford: Oxford University Press (2015). 10.1093/med/9780199811939.001.0001

[29] Kertesz A McCabe P. Recovery patterns and prognosis in aphasia. Brain. (1977) 100:1–18. 10.1093/brain/100.1.1 861709

[30] Levine DN Warach JD Benowitz L Calvanio R. Left spatial neglect: effects of lesion size and premorbid brain atrophy on severity and recovery following right cerebral infarction. Neurology. (1986) 36:362–6. 10.1212/WNL.36.3.362 3951703

[31] Anderlini D Wallis G Marinovic W. Language as a predictor of motor recovery: the case for a more global approach to stroke rehabilitation. Neurorehabil. Neural Repair. (2019) 33:167–78. 10.1177/1545968319829454 30757952

[32] Dalemans R Wade DT van den Heuvel WJ de Witte LP. Facilitating the participation of people with aphasia in research: a description of strategies. Clin. Rehabil. (2009) 23:948–59. 10.1177/0269215509337197 19570814

[33] Jayes M Palmer R. Initial evaluation of the consent support tool: a structured procedure to facilitate the inclusion and engagement of people with aphasia in the informed consent process. Int. J. Speech Lang. Pathol. (2014) 16:159–68. 10.3109/17549507.2013.795999 23826849

[34] van der Vliet R Selles RW Andrinopoulou ER Nijland R Ribbers GM Frens MA. Predicting upper limb motor impairment recovery after stroke: a mixture model. Ann. Neurol. (2020) 87:383–93. 10.1002/ana.25679 31925838 PMC7065018

[35] Watila MM Balarabe SA. Factors predicting post-stroke aphasia recovery. J. Neurol. Sci. (2015) 352:12–8. 10.1016/j.jns.2015.03.020 25888529

[36] Plowman E Hentz B Ellis C Jr. Post-stroke aphasia prognosis: a review of patient-related and stroke-related factors. J. Eval. Clin. Pract. (2012) 18:689–94. 10.1111/j.1365-2753.2011.01650.x 21395923

[37] Kiran S Thompson CK. Neuroplasticity of language networks in aphasia: advances, updates, and future challenges. Front. Neurol. (2019) 10:295. 10.3389/fneur.2019.00295 31001187 PMC6454116

[38] Trupe LA Varma DD Gomez Y Race D Leigh R Hillis AE. Chronic apraxia of speech and Broca's area. Stroke. (2013) 44:740–4. 10.1161/STROKEAHA.112.678508 23362082 PMC3620682

[39] Payabvash S Kamalian S Fung S Wang Y Passanese J Kamalian S. Predicting language improvement in acute stroke patients presenting with aphasia: a multivariate logistic model using location-weighted atlas-based analysis of admission CT perfusion scans. AJNR Am. J. Neuroradiol. (2010) 31:1661–8. 10.3174/ajnr.A2125 20488905 PMC3640318

[40] Gerstenecker A Lazar RM. Language recovery following stroke. Clin. Neuropsychol. (2019) 33:928–47. 10.1080/13854046.2018.1562093 30698070 PMC8985654

[41] Strand EA Duffy JR Clark HM Josephs K. The apraxia of speech rating scale: a tool for diagnosis and description of apraxia of speech. J. Commun. Disord. (2014) 51:43–50. 10.1016/j.jcomdis.2014.06.008 25092638 PMC4254321

[42] Clark HDJ Strand E Josephs JR. Revisions to the apraxia of speech rating scale. In: Conference on Motor Speech. Newport Beach, CA (2016).

[43] Wambaugh JL Bailey DJ Mauszycki SC Bunker LD. Interrater reliability and concurrent validity for the apraxia of speech rating scale 3.0: application with persons with acquired apraxia of speech and aphasia. Am. J. Speech Lang. Pathol. (2019) 28:895–904. 10.1044/2018_AJSLP-MSC18-18-0099 31306600

[44] Lindström E Werner C. A-ning: neurolingvistisk afasiundersökning. Stockholm: Ersta högsk (1995).

[45] Lindström E Werner C. A-ning: neurolingvistisk afasiundersökning. Standardisering. Stockholm: Ersta sjukhus (2000).

[46] Kaplan E Weintraub S Goodglass H. Boston Naming Test. Austin, TX: Pro-Ed (2001).

[47] Harry A Crowe SF. Is the Boston naming test still fit for purpose? Clin. Neuropsychol. (2014) 28:486–504. 10.1080/13854046.2014.892155 24606169

[48] Tallberg IM. The Boston naming test in Swedish: normative data. Brain Lang. (2005) 94:19–31. 10.1016/j.bandl.2004.11.004 15896380

[49] Josephs KA Duffy JR Strand EA Machulda MM Senjem ML Master AV. Characterizing a neurodegenerative syndrome: primary progressive apraxia of speech. Brain. (2012) 135(Pt 5):1522–36. 10.1093/brain/aws032 22382356 PMC3338923

[50] Botha H Duffy JR Strand EA Machulda MM Whitwell JL Josephs KA. Nonverbal oral apraxia in primary progressive aphasia and apraxia of speech. Neurology. (2014) 82:1729–35. 10.1212/WNL.0000000000000412 24727315 PMC4032207

[51] Fugl-Meyer AR Jääskö L Leyman I Olsson S Steglind S. The post-stroke hemiplegic patient. 1. A method for evaluation of physical performance. Scand. J. Rehabil. Med. (1975) 7:13–31. 1135616

[52] Duncan PW Propst M Nelson SG. Reliability of the Fugl-Meyer assessment of sensorimotor recovery following cerebrovascular accident. Phys. Ther. (1983) 63:1606–10. 10.1093/ptj/63.10.1606 6622535

[53] Gladstone DJ Danells CJ Black SE. The Fugl-Meyer assessment of motor recovery after stroke: a critical review of its measurement properties. Neurorehabil. Neural Repair. (2016) 16:232–40. 10.1177/154596802401105171 12234086

[54] Woodbury ML Velozo CA Richards LG Duncan PW Studenski S Lai SM. Dimensionality and construct validity of the Fugl-Meyer assessment of the upper extremity. Arch. Phys. Med. Rehabil. (2007) 88:715–23. 10.1016/j.apmr.2007.02.036 17532892

[55] Woodbury ML Velozo CA Richards LG Duncan PW. Rasch analysis staging methodology to classify upper extremity movement impairment after stroke. Arch. Phys. Med. Rehabil. (2013) 94:1527–33. 10.1016/j.apmr.2013.03.007 23529144

[56] Hope TMH Friston K Price CJ Leff AP Rotshtein P Bowman H. Recovery after stroke: not so proportional after all? Brain. (2019) 142:15–22. 10.1093/brain/awy302 30535098 PMC6308308

[57] Narayan Arya K Verma R Garg RK. Estimating the minimal clinically important difference of an upper extremity recovery measure in subacute stroke patients. Top. Stroke Rehabil. (2011) 18(Supp. 1):599–610. 10.1310/tsr18s01-599 22120029

[58] Basilakos A Smith KG Fillmore P Fridriksson J Fedorenko E. Functional characterization of the human speech articulation network. Cereb. Cortex. (2018) 28:1816–30. 10.1093/cercor/bhx100 28453613 PMC5907347

[59] Tourville JA Nieto-Castanon A Heyne M Guenther FH. Functional parcellation of the speech production cortex. J. Speech Lang. Hear. Res. (2019) 62:3055–70. 10.1044/2019_JSLHR-S-CSMC7-18-0442 31465713 PMC6813033

[60] Pennati GV Plantin J Carment L Roca P Baron JC Pavlova E. Recovery and prediction of dynamic precision grip force control after stroke. Stroke. (2020) 51:944–51. 10.1161/STROKEAHA.119.026205 31906829

[61] Dronkers NF. A new brain region for coordinating speech articulation. Nature. (1996) 384:159–61. 10.1038/384159a0 8906789

[62] New AB Robin DA Parkinson AL Duffy JR McNeil MR Piguet O. Altered resting-state network connectivity in stroke patients with and without apraxia of speech. Neuroimage Clin. (2015) 8:429–39. 10.1016/j.nicl.2015.03.013 26106568 PMC4473263

[63] Wortman-Jutt S Edwards D. Poststroke aphasia rehabilitation: why all talk and no action? Neurorehabil. Neural Repair. (2019) 33:235–44. 10.1177/1545968319834901 30900528

[64] Zhao LR Willing A. Enhancing endogenous capacity to repair a stroke-damaged brain: an evolving field for stroke research. Prog. Neurobiol. (2018) 163–164:5–26. 10.1016/j.pneurobio.2018.01.004 29476785 PMC6075953

[65] Cramer SC Riley JD. Neuroplasticity and brain repair after stroke. Curr. Opin. Neurol. (2008) 21:76–82. 10.1097/WCO.0b013e3282f36cb6 18180655

[66] Raffin E Hummel FC. Restoring motor functions after stroke: multiple approaches and opportunities. Neuroscientist. (2018) 24:400–16. 10.1177/1073858417737486 29283026

[67] Crinion J Holland AL Copland DA Thompson CK Hillis AE. Neuroimaging in aphasia treatment research: quantifying brain lesions after stroke. Neuroimage. (2013) 73:208–14. 10.1016/j.neuroimage.2012.07.044 22846659 PMC3534842

[68] Hartwigsen G Saur D. Neuroimaging of stroke recovery from aphasia – insights into plasticity of the human language network. NeuroImage. (2019) 190:14–31. 10.1016/j.neuroimage.2017.11.056 29175498

[69] Stewart JC Cramer SC. Genetic variation and neuroplasticity. J. Neurol. Phys. Ther. (2017) 41:S17–S23. 10.1097/NPT.0000000000000180 28628592 PMC5477674

[70] Nguyen VA Crewther SG Howells DW Wijeratne T Ma H Hankey GJ. Acute routine leukocyte and neutrophil counts are predictive of poststroke recovery at 3 and 12 months poststroke: an exploratory study. Neurorehabil. Neural Repair. (2020) 34:844–55. 10.1177/1545968320948607 32940147

[71] Crosson B Rodriguez AD Copland D Fridriksson J Krishnamurthy LC Meinzer M. Neuroplasticity and aphasia treatments: new approaches for an old problem. J. Neurol. Neurosurg. Psychiatry. (2019) 90:1147–55. 10.1136/jnnp-2018-319649 31055282 PMC8014302

[72] Price CJ Seghier ML Leff AP. Predicting language outcome and recovery after stroke: the PLORAS system. Nat. Rev. Neurol. (2010) 6:202–10. 10.1038/nrneurol.2010.15 20212513 PMC3556582

[73] Fridriksson J Guo D Fillmore P Holland A Rorden C. Damage to the anterior arcuate fasciculus predicts non-fluent speech production in aphasia. Brain. (2013) 136(Pt 11):3451–60. 10.1093/brain/awt267 24131592 PMC3808690

[74] Basilakos A Fillmore PT Rorden C Guo D Bonilha L Fridriksson J. Regional white matter damage predicts speech fluency in chronic post-stroke aphasia. Front. Hum. Neurosci. (2014) 8:845. 10.3389/fnhum.2014.00845 25368572 PMC4201347

[75] Birchenall J Teremetz M Roca P Lamy JC Oppenheim C Maier MA. Individual recovery profiles of manual dexterity, and relation to corticospinal lesion load and excitability after stroke - a longitudinal pilot study. Neurophysiol. Clin. (2019) 49:149–64. 10.1016/j.neucli.2018.10.065 30391148

[76] Feng W Wang J Chhatbar PY Doughty C Landsittel D Lioutas VA. Corticospinal tract lesion load: an imaging biomarker for stroke motor outcomes. Ann. Neurol. (2015) 78:860–70. 10.1002/ana.24510 26289123 PMC4715758

[77] Bohland JW Bullock D Guenther FH. Neural representations and mechanisms for the performance of simple speech sequences. J. Cogn. Neurosci. (2010) 22:1504–29. 10.1162/jocn.2009.21306 19583476 PMC2937837

[78] Miller HE Guenther FH. Modelling speech motor programming and apraxia of speech in the DIVA/GODIVA neurocomputational framework. Aphasiology. (2020). 10.1080/02687038.2020.1765307. [Epub ahead of print]. PMC8183977 34108793

[79] Glize B Bigourdan A Villain M Munsch F Tourdias T de Gabory I. Motor evoked potential of upper-limbs is predictive of aphasia recovery. Aphasiology. (2018) 33:105–20. 10.1080/02687038.2018.1444137

[80] Blumstein SE Baum SR. Neurobiology of Speech Production: Perspective from Neuropsychology and Neurolinguistics. New York, NY: Elsevier Inc (2016). p. 689–99. 10.1016/B978-0-12-407794-2.00055-9

[81] Difrancesco S Pulvermüller F Mohr B. Intensive language-action therapy (ILAT): the methods. Aphasiology. (2012) 26:1317–51. 10.1080/02687038.2012.705815

[82] Wolf SL Winstein CJ Miller JP Taub E Uswatte G Morris D. Effect of constraint-induced movement therapy on upper extremity function 3 to 9 months after stroke: the EXCITE randomized clinical trial. JAMA. (2006) 296:2095–104. 10.1001/jama.296.17.2095 17077374

